# Modelling *knowlesi *malaria transmission in humans: vector preference and host competence

**DOI:** 10.1186/1475-2875-9-329

**Published:** 2010-11-16

**Authors:** Laith Yakob, Michael B Bonsall, Guiyun Yan

**Affiliations:** 1 Program in Public Health, College of Health Sciences, University of California, Irvine, CA 92697, USA; 2 School of Biological Sciences, St. Lucia Campus, University of Queensland, Brisbane 4072, Australia; 3 Mathematical Ecology Research Group, Department of Zoology, University of Oxford, Oxford OX1 3PS, UK; 4 St Peter's College, New Inn Hall Street, Oxford. OX1 2DL, UK

## Abstract

**Background:**

*Plasmodium knowlesi*, a malaria species that normally infects long-tailed macaques, was recently found to be prevalent in humans in Southeast Asia. While human host competency has been demonstrated experimentally, the extent to which the parasite can be transmitted from human back to mosquito vector in nature is unclear.

**Methods:**

Using a mathematical model, the influence of human host competency on disease transmission is assessed. Adapting a standard model for vector-borne disease transmission and using an evolutionary invasion analysis, the paper explores how differential host competency between humans and macaques can facilitate the epidemiological processes of *P. knowlesi *infection between different hosts.

**Results:**

Following current understanding of the evolutionary route of other human malaria vectors and parasites, an increasing human population in knowlesi malaria endemic regions will select for a more anthropophilic vector as well as a parasite that preferentially transmits between humans. Applying these adaptations, evolutionary invasion analysis yields threshold conditions under which this macaque disease may become a significant public health issue.

**Conclusions:**

These threshold conditions are discussed in the context of malaria vector-parasite co-evolution as a function of anthropogenic effects.

## Background

Although principally a disease of long-tailed and pig-tailed macaques, *Plasmodium knowlesi *has recently been revealed to be a widespread, and potentially life-threatening, malaria infection of humans in Southeast Asia [1]. However, before the public health threat that this disease poses can be assessed, a solid understanding of the fundamental epidemiological processes is needed. Using a simple mathematical model, key components of the disease are explored that are of particular influence in its transmission and yet for which data are lacking, thereby highlighting priority research areas.

Through a series of laboratory experiments, Chin *et al *demonstrated the ability of this parasite to transmit from humans to both simian and human hosts [2]. However, the extent of human host competency under natural conditions remains unclear, giving rise to controversy over whether *P*. *knowlesi *can be classed as the fifth aetiological agent of human malaria [3-5]. Implicit to the epidemiological understanding of this disease is a formalized description of its transmission dynamics. Mathematical methods of Macdonald [6] are, therefore, adapted to allow for multiple host species and used to determine the significance of human host competency in disease transmission.

Traditionally, mosquitoes with higher anthropophily (such as *Anopheles gambiae*) are considered more efficient vectors of human malaria [7]. This is because blood meals taken from alternative mammalian hosts reduce the transmission intensity of human disease. Therefore, the second component considered in this analysis is the influence of vector host preference on knowlesi malaria transmission dynamics under the condition that humans are either non-competent (dead-end) or competent hosts.

In the case where humans are competent hosts for *P*. *knowlesi*, evolutionary theory would suggest that selection favours a parasite that has co-evolved with its natural host, and this has important implications for among-species transmission potential. However, the limited immunological data on this pathogen suggests that serial passage of *P*. *knowlesi *through humans can result in increasingly higher parasite densities, until this pathogen reaches life-threatening levels [8]. In this analysis, the consequences of parasite life history that affect infectivity of knowlesi malaria to humans are determined. Using evolutionary invasion analysis, the conditions necessary for a switch of evolutionary strategies in favour of human knowlesi malaria transmission are calculated. Results are discussed in the evolutionary context of all human malarias and the implications to all vector-borne pathogens are described.

## Methods

The dynamics are described by a set of ordinary differential equations, adapted from Smith *et al *(2007) [9] to include multiple hosts (e.g, humans and macaques). The model is partitioned into three equations; one that represents the proportion of infected humans ($\dot{H}$):

(1) $$\frac{d\dot{H}}{dt}=\frac{V}{H}bhc_{VH}\dot{V}(1-\dot{H})-r\dot{H}$$

one that describes the proportion of infected macaques ($\dot{M}$):

(2) $$\frac{d\dot{M}}{dt}=\frac{V}{M}bmc_{VM}\dot{V}(1-\dot{M})-r\dot{M}$$

and finally one that describes the proportion of infected vectors ($\dot{V}$):

(3) $$\frac{d\dot{V}}{dt}=b\left(\dot{H}hc_{HV}+\dot{M}mc_{MV}\right)(\exp^{-gT}-\dot{V})-g\dot{V}$$

where *V*, *H *and *M *are the vector, human and macaque density; *b *is the bite rate; *g *is the daily vector mortality rate; *T *is the extrinsic incubation period; *r *is the rate of mammalian recovery from infection; *c_VH _*and *c_VM _*are the parasite transmission coefficients from vector to human and from vector to macaque respectively; *c_HV _*and *c_MV _*are the parasite transmission coefficients from human to vector and from macaque to vector respectively; *h *and *m *are the proportion of bites that fall on humans and macaques respectively (see below). The vectorial capacity (the capacity of the vector to transmit infection to humans) is determined from:

(4) $$Vc=\frac{\frac{V}{H}b^{2}h\left(hC_{H}+mC_{M}\right)\exp\left(-gT\right)}{g}$$

where, *C_H _*and *C_M _*is the parasite transmission efficacy from human-to-human (the product of *c_HV _*and *c_VH_*) and macaque-to-human (the product of *c_MV _*and *c_VH_*), respectively. The basic reproductive number of malaria, *R_0_*, is simply the product of *Vc *with the average rate of human recovery (1/*r*).

Following Smith et al. (2007) [9], the infected proportions of humans ($\bar{H}$), macaques ($\bar{M}$) and vectors ($\bar{V}$) at equilibrium are:

(5) $$\bar{H}=\frac{R0-1}{R0+bhc_{HV}g^{-1}}$$

(6) $$\bar{M}=\frac{R0-1}{R0+bmc_{MV}g^{-1}}$$

(7) $$\bar{V}=\frac{b\left(\bar{M}mc_{MV}+\bar{H}hc_{HV}\right)}{g+b\left(\bar{M}mc_{MV}+\bar{H}hc_{HV}\right)}\exp(-gT).$$

As noted, *h *and *m *are the proportion of bites that fall on humans and macaques respectively and are calculated as follows:

(8) h=HQ/(M(1−Q)+HQ)

(9) m=M(1−Q)/(M(1−Q)+HQ).

In this way, the proportion of bites made on alternative hosts accounts for vector preference for human blood (0≤*Q*≤1) as well as the relative host densities. Using these equations, the relationship between vectorial capacity and vector preference for human blood is examined for systems in which humans are either dead-end (*C_H _*= 0) or competent hosts (*C_H _*> 0).

While the parasite transmission efficacy between vector and human hosts is well established for true human malarias, data for knowlesi parasite transmission are lacking. Using classic parameter values that have been recorded for human malarias (daily mosquito mortality of 0.15 [10], extrinsic incubation period of 10 days, daily bite rate of 0.25 and transmission efficacy from human to vector to human of 0.05 [11]) the infection dynamics are simulated for variable parasite infectivity as a function of alternative transmission routes (via macaque or via human). There are two opposing mechanisms by which infectivity might be expected to differ according to transmission routes: 1) given it has co-evolved with macaques, more effective parasite transmission from macaque to mosquito to human might be expected, but 2) a parasite that has already been passaged through a human immune system might be expected to have greater infectivity to a secondary human. Therefore, the dynamics of both alternative assumptions are presented.

The ultimate aim of this study is to understand what conditions an alternative vector could evolve (invade). Evolutionary invasion analysis explores where the resident vector, human, macaque populations are extant and at equilibrium. Novel strategies invade from rare (*V_novel _*density is very small compared to the resident vector).

## Results

**Figure 1:**
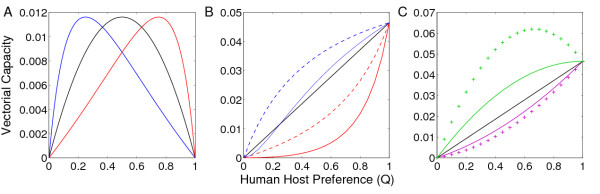
**(**A**) Vectorial Capacity as a function of human host preference when humans are dead-end hosts (i**.e. *C_H _*= 0). The black line corresponds with equal human and macaque density (*H*:*M*); blue with *H*:1/3*M*; red with *H*:3*M*. (**B**) Vectorial Capacity as a function of human host preference when humans are competent hosts. Colours correspond with (**A**), broken lines denote vectors that are 'exclusive' in their host choice and solid lines denote 'mixed' preferences. (**C**) The effect of variable parasite infectivity as a function of its transmission route. Purple corresponds to a parasite that transmits human to mosquito to human more effectively (line, C_H_:1/2C_M_; cross markers C_H_:1/4C_M_), and green corresponds to a parasite that transmits macaque to mosquito to human more effectively (line, C_H_:2C_M_; cross markers C_H_:4C_M_). Unless stated otherwise we use *H*:10*V*; *H*:*M*; *C_H_*:*C_M _*(= 0.05); *b *= 0.25; *g *= 0.15 and *T *= 10.

In the situation where humans are non-competent (dead-end) hosts, vector preference for human hosts has a non-linear relationship with the vectorial capacity (Figure 1A). Intuitively, at the two extremes in human host preference (*Q *= 0, *Q *= 1), a zoonosis cannot persist in humans. In this system, *Plasmodium knowlesi *transmission in humans is dependent on relative mammalian host densities, with the greatest detriment to human health experienced when there is an even distribution of bites between both humans and macaques.

On analysing the dynamics of a system in which humans are competent hosts, the necessity of further scrutinizing the definition of 'host preference' becomes apparent. Under equivalent host preference (*Q *= 0.5), one extreme scenario is that each individual vector exhibits identically indiscriminate host preference ('mixing vectors') while the alternative extreme entails half of the vectors always choosing one host species over the other ('exclusive vectors'). This latter scenario would mirror the dynamics of a traditional human malaria system because a mosquito that only bites humans cannot be infected by macaques. In general, mosquitoes that are exclusive in their host preference are more effective vectors of malaria (Figure 1B).

The relationship between host preference and vectorial capacity is highly dependent on the parasite response to alternative transmission routes (Figure 1C). Consistent with current understanding of human malaria transmission, results show that an exclusively anthropophilic mosquito (*Q *= 1) is usually the optimum vector of malaria in humans when the pathogen no longer requires an alternative mammalian host to complete its lifecycle. However, a special case arises whereby the parasite pervades the human population more readily when transmitted by a generalist vector (Figure 1C). For a generalist mosquito to be a superior malaria vector, the following condition must be satisfied:

(10) $$\frac{C_{H}}{C_{M}}<\frac{Q}{Q+1}$$

Parasite transmission from macaque-to-humans must be more than twice as efficient as human-to-human transmission in order to favour a generalist vector. Alternatively, in the case of an uneven density of humans and macaques:

(11) $$\frac{C_{H}}{C_{M}}<\frac{HQ}{M(1-Q)+2HQ}$$

Hence, conditions are even more permissive for enhanced transmission despite incomplete anthropophily when there is a greater difference in infectivity and when human population density exceeds that of macaques.

Using evolutionary invasion analysis, the fitness (per capita rate of change of a strategy) dynamics of a novel malaria vector (*V_novel_*) are given by:

(12) $$\begin{array}{l} F=\frac{1}{{\dot{V}}_{novel}}\frac{d{\dot{V}}_{novel}}{dt}=\\ \frac{b^{\prime }\left(\bar{M}m^{\prime }{c^{\prime }}_{MV}+\bar{H}h^{\prime }{c^{\prime }}_{HV}\right)(\exp^{-g^{\prime }T^{\prime }}-{\dot{V}}_{novel})-g^{\prime }{\dot{V}}_{novel}}{{\dot{V}}_{novel}} \end{array}$$

where the primes denote parameters associated with this novel strategy. This strategy has positive fitness if *F *> 0, which occurs if:

(13) $$\begin{array}{l} b^{\prime }\left(\bar{M}m^{\prime }{c^{\prime }}_{MV}+\bar{H}h^{\prime }{c^{\prime }}_{HV}\right)(\exp^{-g^{\prime }T^{\prime }}-{\dot{V}}_{novel})\\ -g^{\prime }{\dot{V}}_{novel}>0 \end{array}.$$

This leads to:

(14) $$b^{\prime }\left(\bar{M}m^{\prime }{c^{\prime }}_{MV}+\bar{H}h^{\prime }{c^{\prime }}_{HV}\right)>\frac{g^{\prime }{\dot{V}}_{novel}}{\left(\exp^{-g^{\prime }T^{\prime }}-{\dot{V}}_{novel}\right)}.$$

This fitness threshold can be made explicit for any of the novel vector strategy parameters. This study examines the vector biting behaviour that will allow an alternative parasite transmission cycle to evolve. The proportion of bites on humans (*h'*) by the novel vector strategy must exceed losses due to mortality and the proportion of bites on macaques:

(15) $$h^{\prime }>\frac{g^{\prime }V_{novel}+{c^{\prime }}_{MV}\bar{M}m^{\prime }\left({\dot{V}}_{novel}-\exp(-g^{\prime }T^{\prime })\right)}{C_{HV}\bar{H}\left(\exp(-g^{\prime }T^{\prime })-{\dot{V}}_{novel}\right)}.$$

The evolutionary stable strategy (ESS) with respect to *h' *(the proportion of bites on humans by the novel strategy) is found from setting the partial derivative of equation 12 with respect to *h' *to equal zero:

(16) $$\frac{\partial F}{\partial h^{\prime }}=\frac{b^{\prime }{c^{\prime }}_{HV}\bar{H}(\exp^{-g^{\prime }T^{\prime }}-{\dot{V}}_{novel})}{{\dot{V}}_{novel}}=0.$$

This can be simplified to show that at the ESS associated with the proportion of bites on humans, the average mosquito lifespan is:

(17) $$\frac{1}{{g^{\prime }}_{ESS}}=\frac{T^{\prime }}{-\ln({\dot{V}}_{novel})}.$$

As noted, the novel vector strategy is rare so, conservatively, vectorial capacity (equation 7) of this malaria vector is:

(18) $$Vc_{novel}=\frac{\frac{1}{H}b{'}^{2}h'\left(h'C{'}_{H}+m'C{'}_{M}\right)\exp\left(-g'T'\right)}{g'}.$$

Successful spread of this malaria pathogen by an alternative vector occurs if *Vc_novel _*>*Vc *which is true if:

(19) $$\begin{array}{l} \frac{\frac{1}{H}b{'}^{2}h'\left(h'C{'}_{H}+m'C{'}_{M}\right)\exp\left(-g'T'\right)}{g'}>\\ \frac{\frac{V}{H}b^{2}h\left(hC_{H}+mC_{M}\right)\exp\left(-gT\right)}{g} \end{array}.$$

Other things equal, spread of the pathogen by the alternative vector occurs if $\frac{1}{H}>\frac{V}{H}$; which is unlikely to be true as the density of resident vectors (*V*) is always likely to exceed the alternative vector (*V*'). Greater bite rates (*b'*), attacks on humans (*h'*) or increased longevity (1/*g'*) (see equation 17) favour greater vectorial capacity.

**Figure 2:**
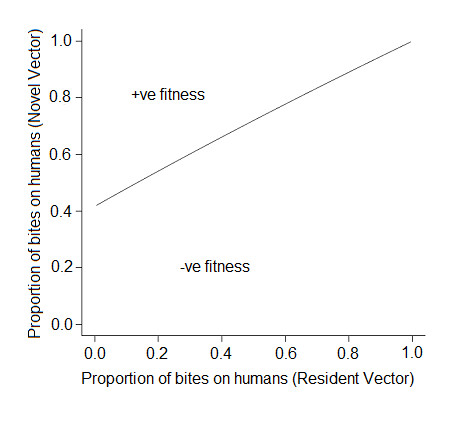
**Fitness boundary of the proportion of bites on humans by a novel vector necessary to exclude the resident vector**. Novel vectors need to achieve a threshold level of bites on humans (at least 40%) in order to evolve (invade). Fitness is determined from equation 12 and represents the per capita growth rate of the novel vector strategy in the presence of alternative vectors and hosts when at equilibrium.

Further, if it assumed that the density of humans (*H*) is constant over the evolutionary ecological changes associated with the vector then the invasion potential of the alternative parasite transmission cycle (via humans) can be evaluated. This is illustrated in Figure 2. Novel strategies are more likely to evolve if the proportion of bites on humans exceeds a critical threshold value (Figure 2). Specifically, if resident vectors of the parasite have equal preference for humans and macaques, novel strategies evolve if they have a greater than 50% preference for human hosts. Similarly, even if malaria vectors have no or low preference for humans (h→0) then the proportion of bites on humans by the novel vector (*h'*) needs to be substantial (greater than 40%) but not necessarily requiring that humans are the preferred host in order for this malaria vector to spread (Figure 2).

## Discussion

Human-to-human transmission of *P. knowlesi *has been shown to occur under experimental conditions [2], and with human populations increasingly encroaching on and supplanting macaque habitat, there is powerful selective pressure for the parasite to switch its natural mammalian host. *Plasmodium vivax*, the most prominent human malaria species in SE Asia and the closest relative to *P*. *knowlesi*, is believed to have a similar evolutionary route [12]. Whether this switch is already occurring in *knowlesi *malaria is currently unclear [13].

Concurrent with a parasite that is potentially switching host due to increased exposure to a human population, specialization of the associated vector might be expected for the same reason. For example, isomorphic members of the *Anopheles gambiae *species complex have diverse feeding patterns, several of which have rapidly co-evolved with the relatively recent anthropogenic practices of agriculture and permanent settlement [14,15]. Speciation, such as that seen in the *Anopheles gambiae *complex, would be expected to benefit the persistence of the knowlesi malaria parasite as it becomes more dependent on the increasingly prevalent human host population. This analysis highlights the critical threshold conditions for a switch in evolutionary stable strategies.

Here, two distinct mechanisms of measuring host selectivity have been described: 'human host preference' is the proportion of bites that fall on humans, averaged over the population of vectors, whereas 'exclusivity' refers to the individual mosquito level of preference. This is an important distinction because when between-individual differences in host choice are taken into account, exclusive mosquitoes normally constitute better vectors of human infection than mixing vectors. For example, when 20% of the total mosquito blood meals are taken from humans, the parasite pervades the human population more effectively if it is the same 20% of mosquitoes always biting humans than if it is all mosquitoes biting humans 20% of the time. This is analogous to the finding that heterogeneous bite rates across a population enhances the persistence of a vector-borne disease, as found by other studies [16-18]. Whereas the heterogeneity was considered in human "attractiveness" or availability previously, in this analysis, heterogeneity in vector preferences is examined. Also important in malaria transmission are heterogeneities in the susceptibility of hosts to infection [19]. Empirical studies have suggested that these sorts of heterogeneities are important in human infection with knowlesi [20]. Once the extent of heterogeneity of macaque infection is characterised, the significance of this addition can be estimated using the framework developed in this study.

Strong anthropophily is normally considered a prerequisite for an effective malaria vector in humans [7]. In this respect, *P. knowlesi *might be expected to differ from true human malarias because its spread to the human population necessitates a generalist vector under the circumstances that humans are dead-end hosts. Interestingly, even if the parasite no longer requires the macaque host to persist, this analysis demonstrates how a generalist mosquito may still prove to be a more effective vector of malaria in humans.

## Conclusions

Accurately assessing the threat to public health that this pathogen poses clearly necessitates extensive empirical work. The problem is confounded further by the fact that there are numerous vector species with extremely variable anthropophily, and many simian host species [21,22]. Even in the simplified description of the system presented here, qualitative differences arose from alternative assumptions of human host competency, emphasizing this to be a critical research area. Additionally, given that most vector-borne human infections are zoonoses, clarifying ambiguous definitions of host preference is also a matter of profound public health importance.

## Competing interests

The authors declare that they have no competing interests.

## Authors' contributions

Model construction and analysis by LY and MBB. Manuscript prepared by LY, MBB and GY. All authors read and approved the final manuscript.
